# A Portable Insole System for Actively Controlled Offloading of Plantar Pressure for Diabetic Foot Care

**DOI:** 10.3390/s25123820

**Published:** 2025-06-19

**Authors:** Pedro Castro-Martins, Arcelina Marques, Luís Pinto-Coelho, Pedro Fonseca, Mário Vaz

**Affiliations:** 1 CIETI, ISEP, Polytechnic of Porto, rua Dr. António Bernardino de Almeida, 4249-015 Porto, Portugal; 2 Faculty of Engineering, University of Porto, rua Dr. Roberto Frias, 4200-465 Porto, Portugal; 3 INEGI, Institute of Science and Innovation in Mechanical and Industrial Engineering, 4200-465 Porto, Portugal; 4 INESC-TEC, Centre for Robotics in Industry and Intelligent Systems, 4200-465 Porto, Portugal; 5 LABIOMEP-UP, Porto Biomechanics Laboratory, Faculty of Sports, University of Porto, rua Dr. Plácido Costa 91, 4200-450 Porto, Portugal

**Keywords:** diabetic foot, health and safety, offloading, plantar pressure, pneumatic insole, ulcers, wearable sensors

## Abstract

**Highlights:**

**What are the main findings?**

- The pneumatic insole can monitor, stabilize and offload plantar pressure in real time.
- Over 91% of measurements are reliable, with up to about 42% pressure reduction.

**What is the implication of the main finding?**

- Strong potential to support foot injury prevention strategies in at-risk populations.

**Abstract:**

Plantar pressure monitoring is decisive in injury prevention, especially in at-risk populations such as people with diabetic foot. In this context, innovative solutions such as pneumatic insoles can be essential in plantar pressure management. This study describes the development of a variable pressure system that promotes the monitoring, stabilization, and offloading of plantar pressure through a pneumatic insole. This research was also intended to evaluate its ability to redistribute plantar pressure, reduce peak pressure in both static and dynamic conditions, and validate its pressure measurements by comparing the results with those obtained from a pedar^®^ insole. Tests were carried out under both static and dynamic conditions, before and after the pressure stabilization process by air cells and the subsequent pressure offloading. During the validation process, methods were used to evaluate the agreement between measurements obtained by the two systems. The results of the static test showed that pressure stabilization reduced pressure on the heel by 32.43%, distributing it to the metatarsals and toes. After heel pressure offloading, the reduction reached 42.72%. In the dynamic test, despite natural dispersion of the measurements, a trend to reduce the peak pressure in the heel, metatarsals, and toes was observed. Agreement analysis recorded 96.32% in the static test and 94.02% in the dynamic test. The pneumatic insole proved effective in redistributing and reducing plantar pressure, with more evident effects in the static test. Its agreement with the pedar^®^ system reinforces its reliability as a tool for measuring and managing plantar pressure, representing a promising solution for preventing plantar lesions.

## 1. Introduction

The foot is a complex and essential structure of the human body that plays an important role in locomotion and balance, supporting the total weight of the body during daily activities [1]. While walking, forces equivalent to about one and a half times a person’s body weight are transmitted through each step [2], thus making the feet susceptible to a series of problems, especially when imbalances occur in the distribution of plantar pressure. Therefore, foot health is essential for general well-being, and any change that generates plantar overload can lead to injury situations [1,2]. This topic is particularly important in the population living with diabetes mellitus, which currently affects about 536.6 million people worldwide and is forecast to affect 642.7 million by the year 2030. One of the most debilitating complications of this disease is the development of diabetic foot, a condition associated with an increased risk of ulceration and amputation in the lower limbs [3].

Several key differences between healthy and diabetic groups underscore the necessity of tailored interventions for individuals with diabetic foot. Healthy individuals typically possess normal skin sensitivity in their feet, enabling them to perceive pain, pressure, and temperature changes effectively. In the diabetic population, peripheral neuropathy, a common condition in this group, leads to a significant reduction in foot skin sensitivity, making patients unable to detect excessive pressures that would normally cause pain. As a result, pressure ulcers form, often in areas of increased plantar pressure, such as the heel and metatarsal areas [4]. According to several studies [5,6], the inability to properly redistribute plantar pressure in the feet exponentially increases the risk of severe ulcerations and subsequent infections, compromising not only immediate health, but also patients’ quality of life over time [5,6,7]. Furthermore, individuals with diabetic foot pathology exhibit significant differences in foot biomechanics and plantar pressure distribution compared to healthy individuals. The loss of protective sensation caused by peripheral neuropathy often leads to altered gait patterns and an inability to detect excessive pressure or impending injury. These changes result in prolonged and uneven plantar loading, particularly in areas such as the heel and metatarsal heads, thereby increasing the risk of skin lesions, ulceration, and infection. Additionally, structural deformities, reduced joint mobility, and impaired microcirculation further compromise foot function and healing capacity. In contrast, healthy individuals retain intact sensory feedback, normal gait mechanics, and effective plantar pressure redistribution during locomotion and standing, which helps prevent tissue damage. These biomechanical and physiological disparities underscore the need for targeted interventions, such as pressure-redistributing insoles, specifically designed for at-risk populations [8,9].

Despite the regular monitoring that can be performed in people with diabetes [10,11], the risk of developing a pressure ulcer in the foot during their active life has a probability that can reach up to 34%, and the level of ulcer recurrence between the first and third year after cure is expected to be 40% and 60%, respectively [12]. Due to these factors, among others, predictions have been made that indicate that worldwide, there is an amputation every 20 s in patients with diabetic foot [13]. This implies a progressive degradation of the patient’s quality of life, as well as imposing an important economic and financial burden on their families and society in general [3].

To mitigate the problems described, this work proposed the development of a system based on an innovative pneumatic insole. The primary aim is to develop and validate a pneumatic insole system capable of monitoring, stabilizing, and offloading plantar pressure, thereby addressing key limitations of existing solutions. To achieve this, the work focuses on four main technical goals: (i) the development of a plantar pressure data acquisition system; (ii) the implementation of a continuous monitoring mechanism that compares real-time pressure values against predefined thresholds; (iii) the design of a closed-loop control strategy for plantar pressure regulation; and (iv) the creation of an active pneumatic insole capable of adjusting its internal pressure in real-time. Additionally, the work further aims to describe the design and fabrication processes of the insole and its electropneumatic control system, evaluate its effectiveness in redistributing and offloading plantar pressures through laboratory-based experimental testing under static and dynamic conditions, and critically analyze the results to identify current limitations and propose future improvements.

This document details the proposed system in Section 3. Here, the methodologies used in the development and manufacturing stages of the pneumatic insole are described, as well as the operation of the electropneumatic control system. In Section 4, to demonstrate the operation of the insole intervention when it is requested to act in the pressure offloading, a set of experimental laboratory tests was developed. The obtained results are described and partially discussed. Section 5 provides an overall discussion and highlights some of the identified limitations. Finally, Section 6 presents the main conclusions and discusses future work.

## 2. Related Work

One of the most debated solutions in the field of pressure ulcer prevention is the use of therapeutic and personalized footwear. Although user-adapted footwear design has advanced significantly in recent decades, many of the models available are passive, meaning they do not actively respond to changes in plantar loads that occur throughout the day [14]. As far as the authors’ knowledge goes, the state of the art on active insoles is scarce. Very recently, system-level approaches have been proposed to regulate plantar pressure distribution according to clinically recommended thresholds, usually based on the most consensual threshold of 200 kPa [5] for the maximum permissible pressure in each area of the foot. These systems are based on magnetorheological fluids [15] and air-powered pneumatic mechanisms [16,17] to control the pressure offloading in cells distributed in an insole or directly on the sole of the user’s shoe [15,16,17].

The work by Hemler et al. [15] presents the development of a plantar pressure relief system integrated into adaptive footwear, designed to prevent ulcers in patients with diabetes. This system uses sensors to continuously monitor pressures and a mechanism with modules containing magnetorheological fluids. When an exciting magnetic field is applied to the fluid, the modules change their stiffness to relieve plantar pressure. However, this system does not act on the toes or midfoot areas [15]. A more recent work by Erel et al. [16], as far as the authors are aware, is the only published work that shares similarities with the solution proposed here; it describes an insole designed to cyclically relieve plantar pressure for patients with diabetic foot. The insole is made of seven air cells distributed throughout the plantar region, four of which alternate controlled inflation and deflation, providing periodic pressure relief in specific areas. This system behaves similarly to an anti-bedsore mattress and aims to mitigate the repetitive stress and excessive load that contributes to the development of plantar ulcers in individuals with diabetic neuropathy [16]. In our previous study [17], we proposed a preliminary concept of an active in-shoe pneumatic insole intended for the prevention of plantar injuries. The findings suggested that an insole with four air-inflated cells (in the toes, metatarsals, lateral-midfoot, and heel regions) would be a viable solution, with air being the most suitable fluid to manage the volume of each cell to redistribute and offload plantar pressure [17]. Although the system proposed by Erel et al. [16] introduces an important approach to pressure relief through cyclic inflation and deflation of air cells, it presents several key limitations. First, the insole was tested in an open-toe sandal, which may compromise accurate pressure measurements, particularly in the forefoot area. This concern has been noted by the authors themselves, who recommend further studies using regular shoes. Second, the study lacks detailed information regarding the pneumatic control unit, including its components, portability, and overall architecture. This omission makes it difficult to fully understand the system’s design or replicate its functionality. Third, the system developed by Erel et al. provides only pre-programmed and uncontrolled cyclic inflation/deflation of the air cells, without incorporating any feedback mechanism or adaptive control. As such, it operates as a passive device, unable to respond to real-time plantar pressure data or clinical risk thresholds. This limits its capacity to deliver targeted, patient-specific pressure relief in dynamically changing conditions.

The challenge, therefore, lies in developing footwear solutions that not only provide greater comfort and reduce peak plantar pressure but also actively monitor and compensate for pressure during gait, thereby mitigating the risk of injury. According to recent research [15,16,17], footwear solutions with dynamic pressure redistribution systems can represent a promising advance in the prevention of diabetic foot injuries. The reported developments are motivated by the understanding that continuous and repetitive loading, particularly high peak pressure, is a major causal factor for foot ulcers, especially in individuals with diabetic neuropathy who may lack protective pain sensation. The aim is to relieve elevated pressures and minimize repetitive stress. The varied efficacy and limitations of existing interventions underscore the need for advanced, dynamic solutions that can actively manage pressure distribution. These efforts to develop active, adaptive, and potentially more user-friendly insoles or footwear clearly indicate a significant trend in research and development aimed at improving prevention strategies.

In addition to the benefits for patients with diabetes in particular, footwear equipped with active plantar pressure offloading systems can bring important advantages in the field of occupational safety and health. Workers who remain on their feet for long periods or who perform repetitive activities are particularly vulnerable to injuries from continuous stress, which not only affects their health but also compromises productivity due to higher absenteeism [18,19,20]. The development of footwear solutions that dynamically offload plantar pressure with real-time monitoring could be a solution to significantly reduce fatigue, increase comfort, and prevent musculoskeletal conditions.

In summary, the field of pressure ulcer prevention is increasingly focusing on the development of therapeutic and personalized footwear that can actively respond to changes in plantar loads. Recent advancements include dynamic pressure redistribution systems, such as air-powered pneumatic mechanisms and magnetorheological fluids, which aim to regulate plantar pressure to recommended thresholds. These systems, integrated into adaptive footwear or insoles, show promise in preventing diabetic foot injuries and mitigating repetitive stress for both patients with diabetes and workers who stand for extended periods. However, the current state of the art is still limited, and further research is needed to fully realize the potential of these active footwear solutions in reducing peak plantar pressure, enhancing comfort, and preventing injuries.

## 3. System Description and Manufacturing Process

To address the above-identified limitations, a novel solution is here proposed: a pneumatic insole system, with active offloading, integrated into a therapeutic shoe certified for diabetic foot care, and designed for both clinical and real-world use.

In this section, the methodology and manufacturing process of the all pneumatic insole system are described, starting with expected requirements (Section 3.1), followed by an overview of the system layout (Section 3.2), the pneumatic insole manufacturing process (Section 3.3), the methodology used for the pneumatic control unit (Section 3.4), and finally, the description of the system operation and interface (Section 3.5).

### 3.1. System Requirements

During the initial design stage, a set of requirements was defined to ensure the effectiveness and performance of the proposed variable pressure system. This is a crucial stage that ensures that operational characteristics cover both the technical specifications and the operating conditions of the device. Table 1 summarizes the most important prerequisites that were considered in the construction and implementation of the prototype of this system, ranging from quantitative to qualitative aspects. The selection of prerequisites was based on information obtained in other studies [4,13,15,16,17] in which various concepts for offloading plantar pressure in the diabetic foot are addressed, including systematic literature reviews [5,6,21], debates with health professionals specialized in diabetic foot pathology, and professionals in the footwear sector.

### 3.2. Variable Pressure System Overview

**Figure 1:**
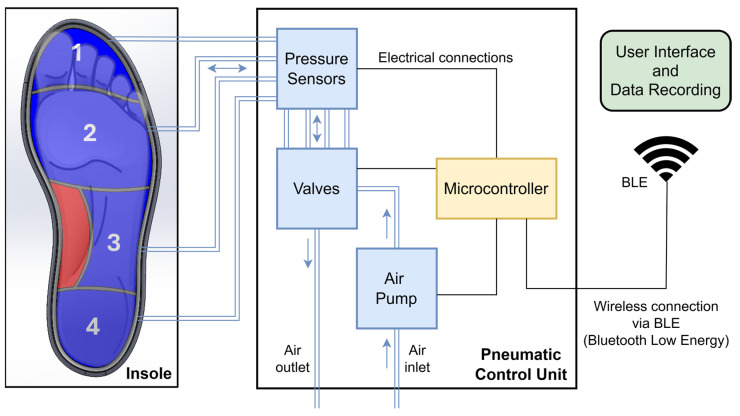
Representative scheme of the variable pressure system. Configuration of the insole air cells in the plantar region of the foot: 1—toes (cell 1); 2—metatarsals (cell 2); 3—lateral-midfoot (cell 3); 4—heel (cell 4); red zone—medial-midfoot (without cell).

The variable pressure system detailed in this study presents itself as an innovative solution for the active management of plantar pressure, integrating pressure sensors with real-time monitoring and pneumatic control. In summary, the system consists of a pneumatic insole equipped with four air cells and a pneumatic control unit, which includes a microcontroller with control electronics, an air pump, a set of pressure sensors, and solenoid valves. User interaction with the system is carried out via a graphical interface with wireless communication based on Bluetooth Low Energy (BLE). Figure 1 shows the representative scheme of the proposed variable pressure system.

The operating principle of the system is based on measuring the return air pressure from the cells to the sensors when the air cells are subject to compression by the areas of the foot they support, as illustrated in Figure 1. Pneumatic pressure sensors constantly measure the pressures applied to the plantar region of the foot during gait and, when pressure levels exceed the previously established threshold, a critical value for the appearance of plantar lesions, the system activates an automatic redistribution mechanism, actuating the solenoid valves to drain and adjust the air pressure in the different cells. An air pump can also be triggered to adjust the volume of air in each of the cells separately when needed. This continuous monitoring and adjustment process ensures that pressure is evenly distributed across the plantar region of the foot, reducing the likelihood of critical pressure points forming.

### 3.3. Insole Design and Manufacturing

**Figure 2:**
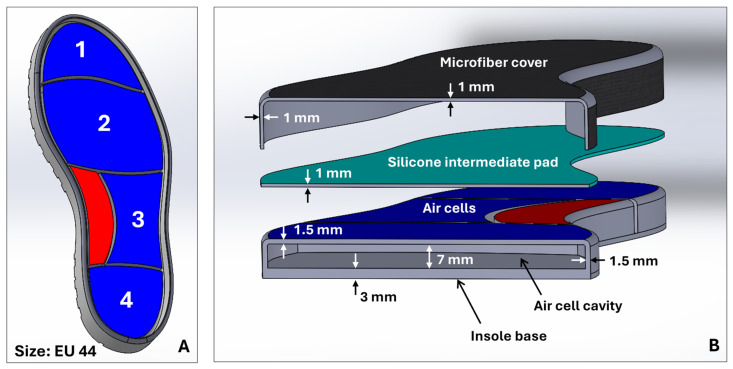
(**A**) Sole concept with layout of the air cells (numbered 1 to 4); (**B**) geometric characteristics of the air cell walls of the insole, silicone intermediate pad, and microfiber upper lining.

The design of the pneumatic insole was developed with 3D modeling tools using computer-aided design (CAD) in SolidWorks (2024-SP5.0, Dassault Systèmes) software. The design process began with the general sizing of the insole, based on the design of a shoe sole designed for a foot size EU44 (Figure 2A). From this model, the insertion zones of the inflatable cells were created. The boundaries of each cell were created as a function of the division of the plantar region of the foot by critical areas of greater clinical interest for plantar pressures and ulcer development [22]: cell 1, toes; cell 2, metatarsals; cell 3, lateral-midfoot; cell 4, heel. Additionally, and considering that the majority of the population has a normal foot type without abnormalities [23], in the medial-midfoot region, an area of the foot less stressed on impact during gait [24], a zone without air cell (red zone, Figure 1 and Figure 2A) was created that serves as an intermediate support zone, providing stability and assisting as a point for additional load distribution. Each cell has been individually designed, with specific dimensions to ensure uniform redistribution of plantar pressure and to meet the biomechanical needs of the foot, with a compromise between the shape of the insole, the anatomical specificities of the foot, and comfort and flexibility. The configuration of the air cells distributed in the plantar region is illustrated in Figure 1 and Figure 2A.

The structure that holds the insole’s air cells was manufactured using a liquid rubber injection-molding process. Figure 2A,B show the concept view for the complete sole with the layout of the air cells and their main geometric features, respectively. It should be noted that the complete insole consists of three components (see Figure 2B): the structure with the air cell cavities, at the base of the insole; a silicone intermediate pad; and a microfiber top coating.

**Figure 3:**
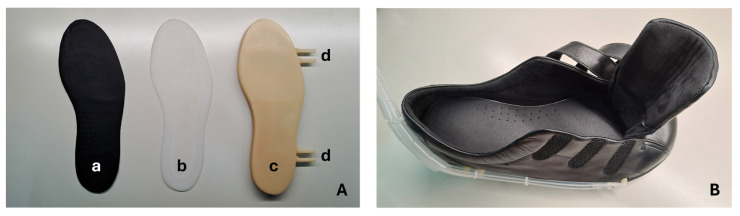
(**A**) Components that make up the pneumatic insole: (a) microfiber, (b) silicone intermediate pad, (c) rubber structure with air cells, and (d) connection points to pneumatic tubes; (**B**) pneumatic insole already inserted into a shoe (Ortomedical, Doctor Karl, EU44) and with the pneumatic connections.

The upper lining of the insole is made of microfiber (onSteam^®^, Grupo Móron, Arnedo, Spain), which, like synthetic leather, is highly breathable and has the capacity to absorb up to eight times its weight in water. This microfiber, in addition to giving a soft finish to the insole, is also an element that promotes stability and prevents the foot from slipping. The silicone intermediate pad, added just below the microfiber, acts as an impact-absorbing layer and minimizes the potential risk of high-pressure points being generated at the locations of the walls that divide the air cells. This element was produced using a liquid silicone pouring mold (Ecoflex™ 00-50, Smooth-On, Macungie, PA, USA), a material that, once cured, is certified as skin safe. Finally, the structure that sits at the base of the insole and accommodates the air cells was produced using a low-pressure liquid rubber (VytaFlex™ 40, Smooth-On, Macungie, PA, USA) injection-molding process. For this process, molds were previously created by a high-resolution fused deposition modeling (FDM) 3D printing technique, after which the liquid rubber was injected, and the respective curing process began. Table 2 provides details of the main characteristics of the liquid rubber and silicone used in the molding processes. Figure 3 shows the pneumatic insole produced and the shoe (Ortomedical, Doctor Karl, Vila do Conde, Portugal) into which it will be inserted. This shoe is certified as a medical device for diabetic foot, including, its inner lining and the upper coating (onSteam^®^ microfiber, Grupo Móron, Arnedo, Spain) of the insole are treated with silver ions, elements that give it antibacterial properties and minimize the possibility of odors inside the shoe.

### 3.4. Pneumatic Control Unit

The pneumatic control unit is divided into two parts that operate together: the control electronics, and the electropneumatic element. At the core of the electropneumatic element are the solenoid valves (0520D; 12 V DC; normally closed; FSPump), responsible for controlling the air inlet and outlet of each cell individually. The four inflatable air cells, individually connected to the control unit by 4 mm diameter silicone tubes, are managed by the control electronics, which automatically regulate the internal pressure by opening and closing the solenoid valves. These solenoid valves, together with pneumatic pressure sensors (XGZP6847D001MPG; 1 MPa; CFSensor) and a mini air pump (3202PM; 12 V DC; Makeblock), allow continuous monitoring and dynamic adjustment of the pressure, ensuring effective redistribution of air through the various areas of the insole. Although the pressure sensors come pre-calibrated from the factory, their measurements were further evaluated through a gauging process. Compressed air was applied, in various pressure ranges, and compared with the reference values of a precision pressure gauge. This process made it possible to confirm the quality of the measurements obtained and ensure reliability within the manufacturer’s stated error margin (±2.5% FSS). Additionally, the pressure sensors used in the system also measure temperature. This functionality allows to estimate the approximate temperature of the foot plantar region, which may be useful for complementary thermal monitoring, by measuring the temperature of the air returning from the inflatable air cells to the sensor.

**Figure 4:**
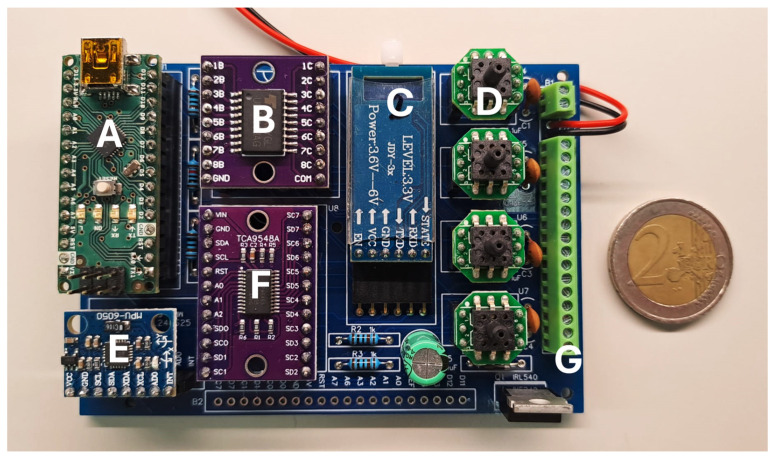
Printed circuit board for control electronics, consisting of: (**A**) microcontroller; (**B**) integrated circuit with power transistors; (**C**) BLE module; (**D**) pressure sensors; (**E**) IMU module; (**F**) I2C multiplexer for communication with peripherals; (**G**) connector bar.

A printed circuit board (PCB) was specifically designed for the system’s control electronics (Figure 4). In addition to securely housing the pressure sensors, this PCB integrates three key components: a microcontroller (based on the ATmega328 chip), a power transistor module (ULN2803A), and a BLE wireless communication module (JDY34). The microcontroller operates the system’s algorithm, which is responsible for processing the readings from the pressure sensors and sending commands to the higher-power peripheral elements through the transistor module, such as the mini-pump and the solenoid valves. Wireless communication via the BLE module facilitates interaction between the system and the user, which is carried out via a mobile application (Serial Bluetooth Terminal, version 1.48) on a smartphone, allowing the user to monitor and intervene with the system in real time. Additionally, an inertial measurement unit (IMU) (MPU6050) was integrated into the PCB to provide acceleration and orientation measurements. This additional information helps the microcontroller determine optimal timing for system adjustments, such as modifying air volume in the cells when the user pauses momentarily while walking. Together, these components ensure coordinated and efficient operation of the system, ensuring active redistribution of plantar pressure to minimize high-pressure events and increase safety.

**Figure 5:**
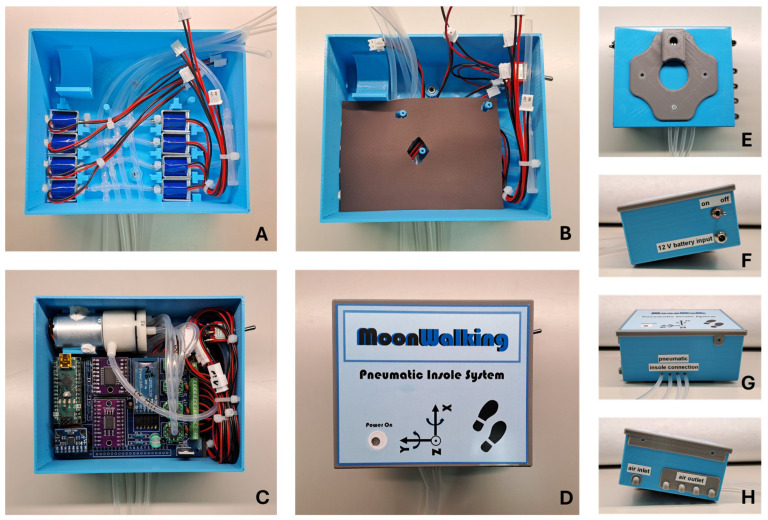
Assembly process of the pneumatic control unit: (**A**) Solenoid valves with derivations and connection to silicone tubes; (**B**) insulation between PCB and solenoid valves; (**C**) PCB, air pump and connections; (**D**) front view of the closed box; (**E**) rear view with waist fastening clip; (**F**) right side view with battery connector and switch; (**G**) lower view with connecting tubes to the insole; (**H**) left side view with one inlet and four air outlets.

Figure 5 shows the different phases of the pneumatic control unit assembly process, using the components mentioned above and through 3D printing processes. To ensure portability, this unit can be powered by a battery (12 V DC; 18650 Li-ion; 3S1P; 9900 mAh). The control unit’s support box was 3D printed (in PLA by the FDM process) and designed so that it can be easily carried on the user’s waist. Once attached to the waist, the silicone tubes go down the leg to the shoe, where they connect to the insole. This design, although still preliminary, facilitates access to electronic and pneumatic components, without compromising the user’s natural mobility to carry out any gait tests.

### 3.5. Operation and Interface

The proposed variable pressure system is prepared to operate in an automated way, based on a dedicated algorithm that responds in real-time to changes in plantar pressures throughout the gait. Pneumatic pressure sensors continuously monitor and record measurements of pressure values with a frequency of 15 Hz. The algorithm processes these measurements and, based on a predefined pressure threshold, sends commands to the exhaust solenoid valves to adjust the volume of air in the inflatable cells. This real-time response is based on the plantar pressure monitoring process, whereby simultaneous measurements are acquired from all four cells in the plantar region with a sampling rate of 15 Hz; that is, the system records 60 samples per second (15 for each cell/region per second). Using this data, and based on the programmed algorithm, the pneumatic control system acts to regulate the pressure in the air cells. This process can take up to 100 ms (from pressure threshold detection to offloading response) and can be executed as many times as necessary until the desired threshold is reached, or until the air volume of a given cell reaches the minimum level for it to remain functional. This method aims to redistribute pressure through the four strategic zones of the insole (heel, lateral-midfoot, metatarsals, and toes) whenever a pressure pattern higher than the 200 kPa threshold (or other pre-configured) is identified, known as the critical point of overload for the appearance of plantar lesions in people with diabetic foot [5,21].

**Figure 6:**
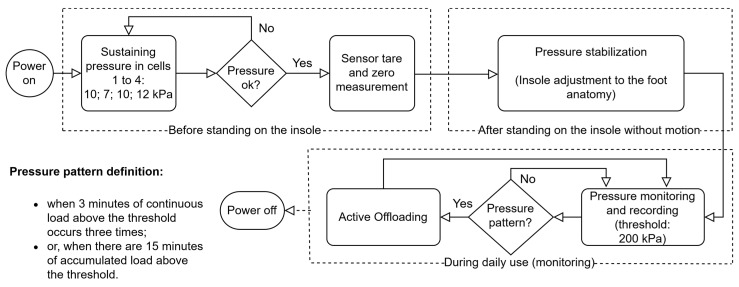
Variable pressure system operation algorithm based on a critical pressure threshold.

The objective is that the pressure redistribution promotes the offloading at points where high-pressure peaks originate, thus standardizing the pressure throughout the plantar region. However, if it is impossible for the system to offload pressure below 200 kPa, other approaches can be used. For example, the pressure in the regions where the highest-pressure peaks originate can be reduced by 25% [21], an alternative also used in plantar pressure reduction strategies in diabetic foot. It is worth mentioning that there are healthy people without risk of injury who sometimes register plantar pressure values above the 200 kPa threshold [5]. In any case, so that the variable pressure system can be as comprehensive as possible, the pressure threshold can be configured and adapted to the user’s real needs. For example, in the case of diabetic foot, setting a certain threshold may be recommended by the patient’s attending physician. Figure 6 shows a flowchart that illustrates the mode of operation of the algorithm for the 200 kPa threshold, the most consensual, although other thresholds can be configured.

As shown in Figure 6, one of the key functionalities is the initial pressure adjustment process to inflate each cell of the insole. When the system is turned on, each cell is pressurized with an adequate sustaining pressure: toes, 10 kPa; metatarsals, 7 kPa; lateral-midfoot, 10 kPa; heel, 12 kPa. These values were obtained for a user with a mass of 79 kg, based on empirical tests, and are adequate to avoid the total sinking of the cells. Comfort and some cushioning, air volume and area of each cell (shoe size EU44: toes, 28 cm^2^; metatarsals, 74 cm^2^; lateral-midfoot, 36 cm^2^; heel, 34 cm^2^) were also considered, as well as an approximation of the distribution of body weight across the different zones of a normal foot when static (toes, 10%; metatarsals, 30%; midfoot, 10%; heel, 50% [25]).

After the initial phase of adjusting the sustaining pressure of the cells, the measurement zero for the sensors is recorded. Once the user is standing on the shoe, the system performs a pressure stabilization process to ensure an even distribution of air in the four inflatable cells of the insole. This adjustment aims to adapt the system to the specific anatomy of the user’s foot while they are still standing on the insole, before starting to walk. This step allows any high-pressure points to be dissipated, maintaining a balanced redistribution of pressures throughout the plantar region of the user’s foot. During this process, the air inlet solenoid valves open, bringing the four cells into communication, causing the user’s body weight to promote airflow and the volume in each cell to be automatically adjusted. This initial balance promotes adaptation to the anatomy of the foot and ensures that no area suffers an overload or excessive pressure, preventing potential discomfort and injury.

**Figure 7:**
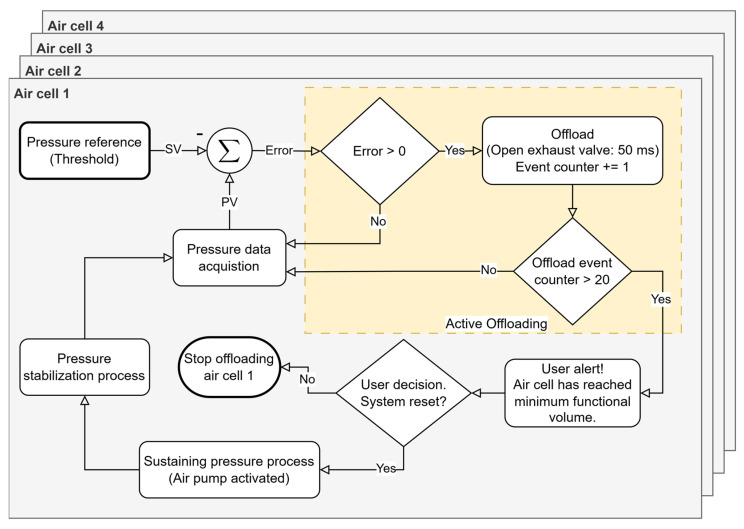
Independent control loop for each air cell pressure regulation (represented by overlaid squares). The pressure reference is based on clinical guidelines, but it can be independently adjusted and personalized for each foot or patient condition. SV, setpoint value; PV, process value.

The pressure offloading process in each air cell is carried out by the exhaust solenoid valves, which drain small fractions of the volume of air present in the inflatable cells in a controlled manner until a pressure value is reached as close as possible to the recommended level. On the other hand, the air pump acts when it is necessary to inject air to restore pressure in a certain cell. This dynamic adjustment happens without direct user intervention, which makes the system autonomous and responsive. Redistribution (pressure offloading from a given region) occurs whenever the system detects a pattern of abnormal pressures (based on the algorithm in Figure 6), ensuring that air is relocated in a way that minimizes high-pressure points that can lead to the development of pressure ulcers or other injuries. However, for pressure corrections that require it, for example due to the user’s accelerated speed, the system can alert the user to stop walking so it can more accurately correct the air volume of the cells. This procedure is only possible with the support of the data provided by the IMU, which will indicate the user’s state of movement; that is, whether the user is stationary and standing in the same position as during the initial moment of pressure stabilization. It should be noted that the capacity for pressure offload is limited by the volume of air inside each cell. When it is not possible to drain more air (each cell needs a quantity of residual air so that they do not sink and for the sensor to be operational), it will also not be possible to relieve more pressure in that region of the foot. In these cases, the system records this event and issues an alert to inform the user. In summary, our approach enables the real-time definition and monitoring of clinically established pressure thresholds, facilitating targeted offloading in specific plantar regions while concurrently tracking potential pressure increases in adjacent areas. This dynamic regulation ensures a balanced and controlled redistribution of plantar pressure, minimizing the risk of new overload points. This pressure management sub-strategy is illustrated in Figure 7 and represents a subroutine of the “Active Offloading” event shown in Figure 6.

**Figure 8:**
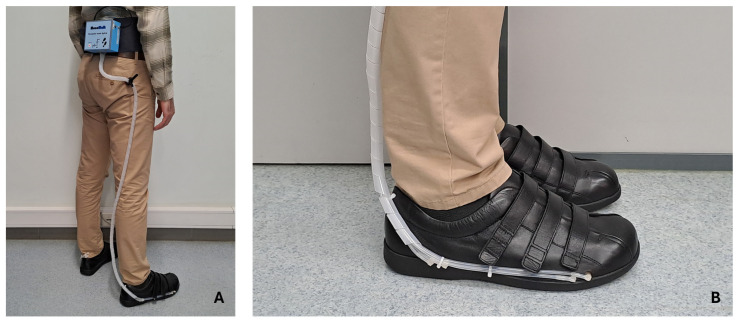
User equipped with the variable pressure system: (**A**) pneumatic control unit attached to the waist; (**B**) final appearance of the shoe (Ortomedical, Doctor Karl, EU44) with pneumatic insole.

In addition to the algorithm handling the data in real-time for its decision-making, it is possible to interact with the system to collect and record data. This is carried out through a mobile application (Serial Bluetooth Terminal, version 1.48) for smartphones, which communicates through BLE. This application allows the user to follow the measurements in real-time and adjust some basic system settings, such as small adjustments in the volume of air in the cells for better comfort, as well as turning the system on and off. As described earlier, the pneumatic control unit will be attached to the user’s waist, with the silicone tubes being connected to the insole that is inserted into the shoe. Figure 8 shows a user equipped with the system.

## 4. Experimental Tests

In this section, the methodology used in the experimental tests (Section 4.1) and in the statistical analysis of the data obtained (Section 4.2) is described. Finally, in Section 4.3, the results obtained in the static and dynamic tests and in the pneumatic insole validation are presented.

### 4.1. Test Protocol

The preliminary tests involved a healthy participant (male, 79 kg) wearing a pair of shoes (Ortomedical, Doctor Karl, EU44) equipped with the pneumatic insole on the right foot (see Figure 8). In these tests, only the right foot was evaluated. However, to provide stability to the participant, a similar pneumatic insole, pressurized and sealed with the pre-established sustaining pressure for each cell (the same for both feet, as indicated in Section 3.5. Operation and Interface) was added to the left shoe. To validate the measurements obtained by the pneumatic insole, the pressures at the interface with the foot were obtained through a commercial system considered the gold standard for the measurement of in-shoe plantar pressure (pedar^®^ insole; sampling rate of 100 Hz; measuring range from 15 kPa to 600 kPa). The variable pressure system collected pressure data from the pneumatic insole at a sampling rate of 15 Hz, with the pressure values rounded to the unit and a measurement uncertainty of ±1 kPa. Its measuring range is from 1 kPa to 1000 kPa with a resolution of 1 kPa.

**Figure 9:**
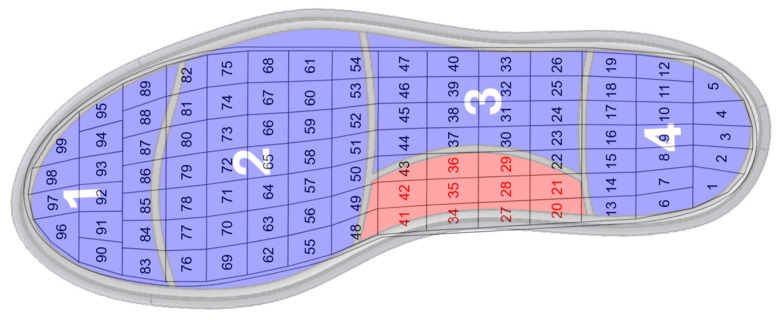
Layout of the pedar^®^ insole sensors superimposed on the pneumatic insole and corresponding to the regions of the foot defined by the air cells. Toes (zone 1): sensors 83 to 99; metatarsals (zone 2): sensors 48 to 82; lateral-midfoot (zone 3): sensors 22 to 26, 30 to 33, 37 to 40, and 43 to 47; Heel (zone 4): sensors 1 to 19.

To measure the participant’s plantar pressure, the pedar^®^ insole was superimposed on the pneumatic insole and both were inserted into the shoe. As the pedar^®^ insole consists of 99 sensors, and to record the pressure for each area of the foot corresponding to each air cell of the pneumatic insole, a division of the sensors was carried out by each of the four regions of the foot. Figure 9 illustrates the correspondence between the measurement areas of both systems.

The participant was evaluated under two test conditions: static and dynamic. For the static tests, the individual remained standing with the shoes on, in a natural way, and with both feet on a rigid surface (laboratory floor), flat and level, for one minute. During this period, plantar pressure data were collected. This procedure was performed with and without pressure stabilization, as previously described in Section 3.5. Operation and Interface. For the dynamic tests, the conditions were like the static tests, although in this case, the participant walked on a level treadmill at a constant speed of 3 km/h for one minute. Additionally, in both test conditions, a simulation of the heel pressure offloading was performed (without considering any specific threshold) with a command sent to the system to trigger the event in the respective air cell. It should be considered that, in both types of tests, time intervals were selected as reasonable time frames for data collection and to verify the effects of plantar pressure; however, these periods have no direct relevance to any clinical requirements. Likewise, the speed selected for the dynamic tests was only chosen to ensure a smooth and paused gait, like a regular walk.

### 4.2. Data Analysis and Statistics

During the analysis of the data obtained, the following terminology was considered to classify some parameters under study: interface pressure (IP): for each air cell of the pneumatic insole, the interface pressure for each region of the foot is measured by the pedar^®^ insole and calculated based on its sensors corresponding to the area of each selected air cell (see Figure 9), summing the pressure data of all sensors and dividing by the number of sensors; average interface pressure (AIP): due to the natural variation of the IP at each measurement instant during the gait cycle and in static mode (although much smaller), the AIP for a given measurement period can be obtained by taking the average of the IP values that are recorded at each instant; peak pressure (PP): The highest reading of a given pressure sensor for each area of the pneumatic insole is designated as the peak pressure of that area.

Non-parametric tests were selected for the statistical analysis to compare paired samples. The Wilcoxon test was used to determine the statistical difference in the pressure data of the interface (pedar^®^ insole) before and after the pressure stabilization procedure in the pneumatic insole, as well as before and after the pressure offloading, during the static and dynamic tests. To ensure a 99% confidence level, a *p*-value < 0.01 was considered statistically significant. The collected data was processed and organized in Excel files, and all statistical analysis was developed in Python (version 3.10).

The conducted tests also aimed to validate the variable pressure system as a viable alternative to existing plantar pressure measurement devices. For this purpose, and considering the pedar^®^ insole as the gold standard in the market, the Bland–Altman method was used to evaluate the level of agreement and equivalence of the pneumatic insole results to assess if it is reliable. For a correct comparison of the two measurement systems, and due to the difference in the sampling rate, a downsampling of the pedar^®^ insole was performed; that is, each measurement obtained by the pneumatic insole corresponds to an average of seven samples of the pedar^®^ insole.

### 4.3. Results

#### 4.3.1. Static Tests

**Figure 10:**
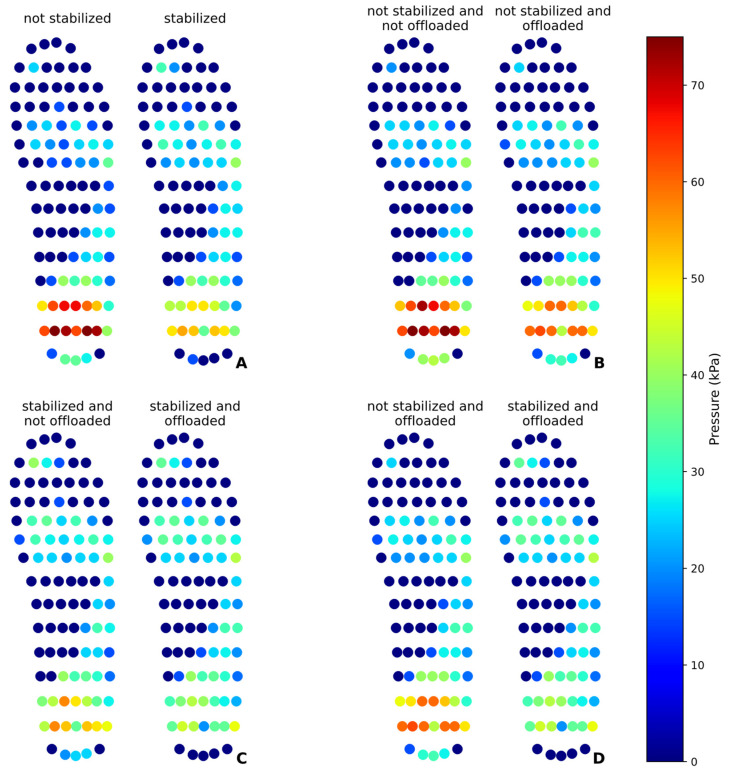
Interface pressure (IP) maps at a time before and after each intervention of the pneumatic insole in the heel region in static mode: (**A**) effect of pressure stabilization; (**B**) effect of pressure offloading without stabilization; (**C**) effect of pressure offloading, previously stabilized; (**D**) effect of stabilization on the pressure offloading process.

The IP and AIP values were analyzed for each region of the pneumatic insole during the tests in static mode. Table 3 presents the AIP values recorded, comparing them under different conditions, before and after the pressure stabilization process, and before and after the heel pressure offloading process. This last pressure offloading procedure was only performed once, and also because the heel is the region identified as the foot area supporting the greatest body weight and, consequently, the region with the greatest pressure. Variations in plantar pressure after the activation of the pressure stabilization and offloading procedures were analyzed. Figure 10 shows an illustration of the evolution of pressure values obtained by the pedar^®^ insole before and after the different interventions of the pneumatic insole under the various test conditions.

Analyzing the data in Table 3, it is possible to observe that all results were considered statistically significant (*p*-value < 0.01), proving that there is an evident difference in the AIP before and after the intervention of the pneumatic insole. The pressure stabilization process proved to be effective as a mechanism for redistributing plantar pressure as a function of foot morphology, while reducing pressure in areas of higher concentration and distributing a portion to other areas. After this process, it was found that the heel (area of higher-pressure concentration) pressure reduced by about 32.43%, and the areas that supported the effect of this reduction were mainly toes and metatarsals, where there was a compensatory increase in pressure. This is an expected behavior, as the body weight is constant and only the pressure is redistributed throughout the plantar region, minimizing any critical points of pressure accumulation. The results obtained are also promising for the pressure offloading procedure, demonstrating effective reduction in AIP following heel pressure offloading intervention, regardless of whether the initial pressure stabilization was performed. When the data is compared without any action having been taken beforehand and after the initial stabilization of the pressure and subsequent offloading, the reduction in pressure on the heel is also very evident. Under these conditions (additional test, marked with ^1^ in Table 3), the reduction came to about 42.72%, where, naturally, the toes and metatarsals once again absorbed part of this portion of reduction. Figure 10 illustrates the pressure reduction results across various test conditions, with the recording of the IP values immediately before and after the respective interventions.

#### 4.3.2. Dynamic Tests

In the dynamic tests, plantar pressure values were recorded with the participant walking on a leveled treadmill at a constant speed of 3 km/h. During walking, the interface pressure was recorded and analyzed for each region of the foot. As this is a dynamic gait test, and as the aim is to evaluate plantar pressures, this analysis only considered the stance phase, not considering the swing phase (foot raised) in which no pressure value is recorded or is very close to zero. Table 4 summarizes the AIP values, highlighting the differences before and after the activation of the pressure offloading mechanism, and its effect was evaluated without and with the action of the initial pressure stabilization by the four air cells of the pneumatic insole. Like the static tests, and for the same reasons, the pressure offloading intervention was only performed once in the heel area.

The data in Table 4 reveal a heterogeneous nature intrinsic to the different phases of human gait movement. The large standard deviation, indicating substantial dispersion in the measurements, reflects the varying plantar pressure amplitudes that occur during different impact and stress moments across foot regions throughout the participant’s walking cycle. Although the test data indicate a trend towards reduced pressure in the intervention area (heel), not all changes recorded in the other areas were considered statistically significant. However, it is important to highlight that the intervention to reduce pressure in the heel produced an effect, especially in the conditions in which the pressure stabilization procedure was performed prior to the pressure offloading event. In this situation, the heel region was offloaded to approximately 24.73% of its previous pressure. This reveals that the stabilization process can be an important factor for better pressure offloading.

**Figure 11:**
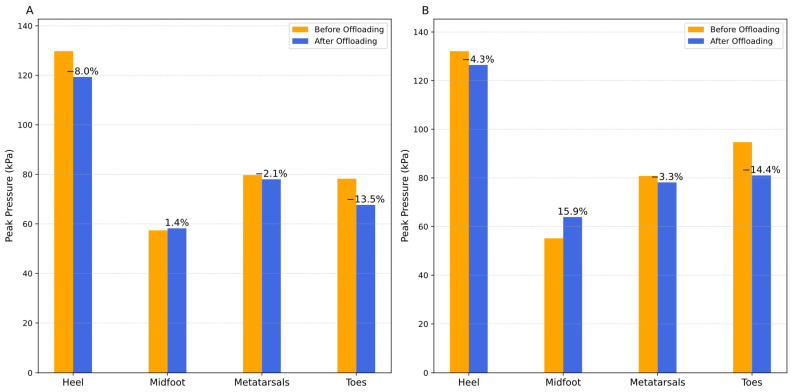
Intensity of plantar peak pressures before and after the heel pressure offloading event, with the percentage recording of their effect on the different regions of the foot in the dynamic test: (**A**) without prior stabilization procedure; (**B**) with prior stabilization.

Naturally, and due to its characteristics, during the movement of human gait, plantar pressure measurements are very dispersed. Thus, in this dynamic mode, it may also be important to assess whether the peak pressure decreased in intensity after the pressure offloading event. Figure 11 reveals this, indicating that there was indeed a reduction in the intensity of the peak pressure in the heel area where the intervention was carried out. In addition to the heel, there was also a reduction in the metatarsal and toe areas. In the midfoot region, there was an increase in peak pressure, and this zone was the one that absorbed the effect of the peak pressure reduction caused in the other regions in the dynamic tests.

#### 4.3.3. Pneumatic Insole Validation

**Figure 12:**
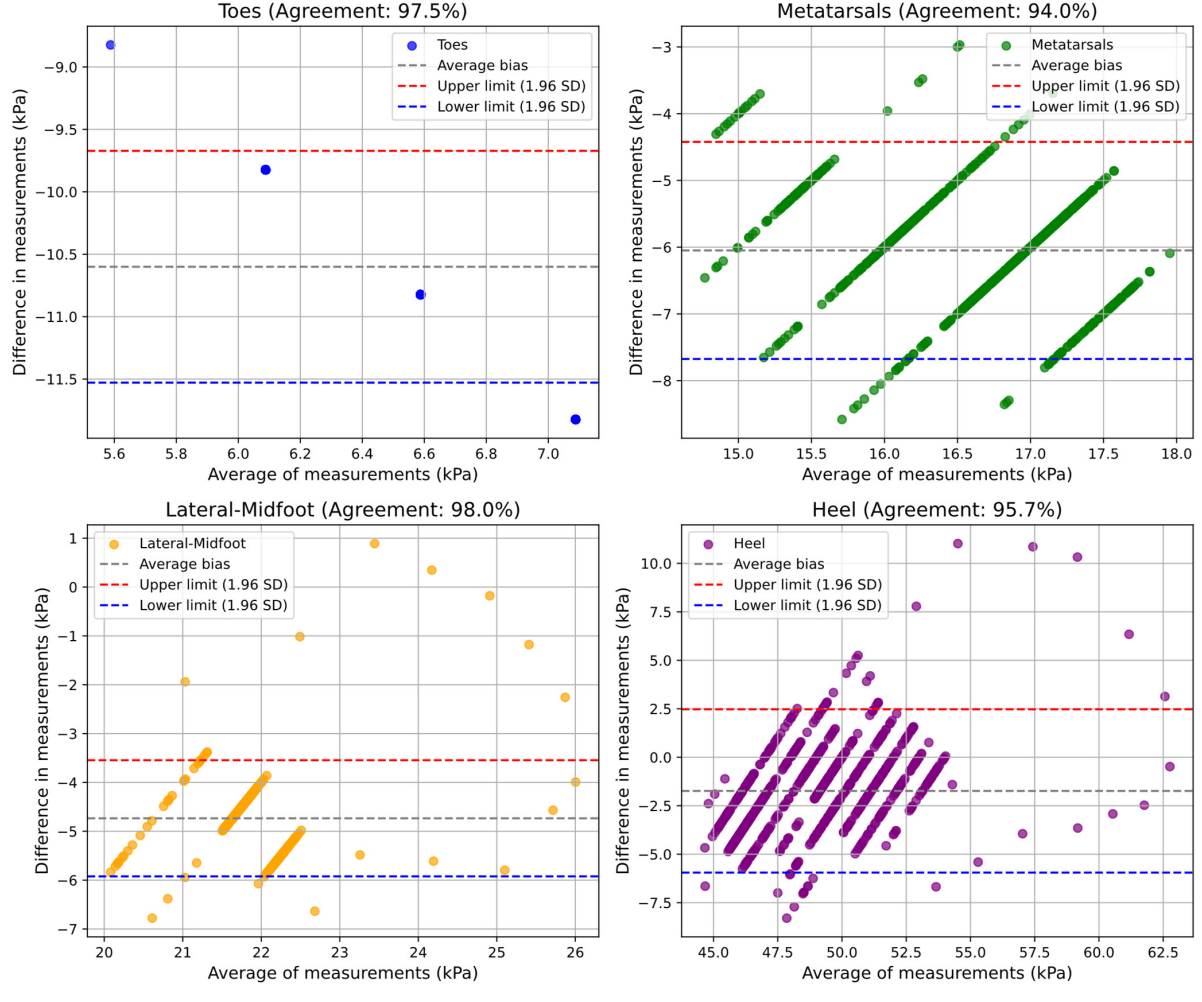
Agreement level evaluation of plantar pressure measurements obtained with the pneumatic insole (pedar^®^ vs. pneumatic insole) during the static test conditions.

**Figure 13:**
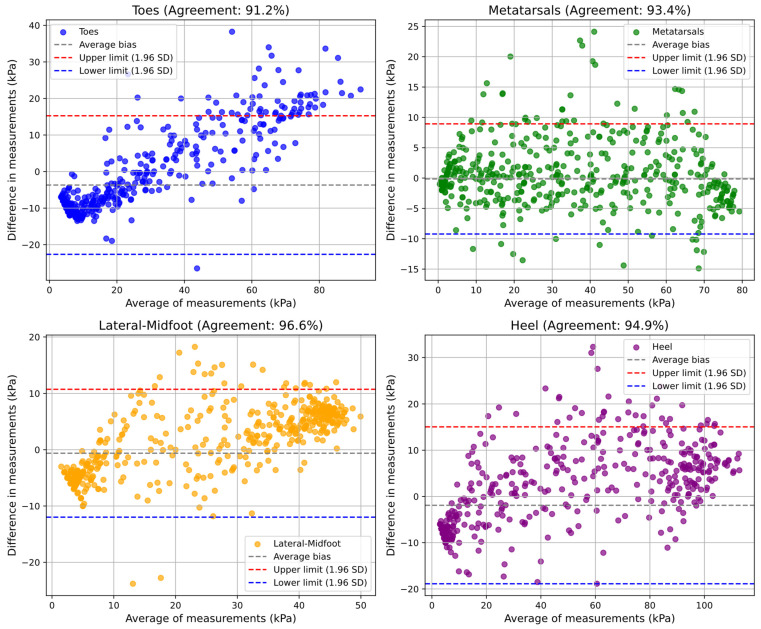
Agreement level evaluation of plantar pressure measurements obtained with pneumatic insole (pedar^®^ vs. pneumatic insole) during the dynamic test conditions.

To validate the pneumatic insole and evaluate its level of reliability in plantar pressure measurements, with a confidence level of 95%, both in static and dynamic conditions, Bland–Altman graphs were produced comparing the pressure values measured by the two systems (pedar^®^ insole vs. pneumatic insole). Figure 12 and Figure 13 present the results obtained in the static and dynamic tests, respectively, for the different regions of the foot, allowing us to verify the level of agreement and the presence of any systematic biases. With the level of confidence conferred by the Bland–Altman method, both in the static and dynamic tests, it can be stated that the pneumatic insole (all of the variable pressure system) shows over 90% agreement with the pedar^®^ insole. Considering all regions of the foot, an overall agreement of 96.32% and 94.02% is obtained for the static and dynamic tests, respectively. For a better interpretation of the graphs, it is important to consider that if the difference between measurements is positive, it means that the pedar^®^ insole has measured pressure values higher than those measured by the pneumatic insole. If the difference is negative, it means that the pedar^®^ insole has measured values lower than those recorded by the pneumatic insole.

For the static test (Figure 12), it is observed that some zones, such as the metatarsals, the midfoot, and the heel, present a particular pattern with several diagonal data bands. Although most of the data are within the limits of agreement, this type of pattern in some zones may indicate that the differences between measurements present a systematic bias dependent on the magnitude of the pressure value; however, the same trend is not always revealed. For example, if at a given time a measurement system tends to overestimate high values and underestimate low values, or vice versa, for the same average value of a given measurement, it can result in differences that do not follow the same trend, increasing or decreasing. It should be noted that in this static test, all the average bias lines present negative values, which indicates that, on average, the pedar^®^ insole measured lower values than the pneumatic insole. However, the lower and upper limits are very close, which is a good indicator. Still analyzing the validation of the pneumatic insole during the static test, it is observed that the toes graph appears to have few data points. In fact, measurements in this region of the foot show very low-pressure values and practically no variation in the two measurement systems, resulting in several equal average values. It is also verified that the higher the pressure value, the greater the difference, with a negative trend, which indicates that the values measured by the pedar^®^ insole are lower than those measured by the pneumatic insole. This is because, effectively, the pedar^®^ insole only measures pressure from 15 kPa, and as the range of measurements in this region of the foot in static mode are mostly below that magnitude, a large proportion of the error is associated with the limited measurement range of the pedar^®^ insole for lower pressures.

For the dynamic test (Figure 13), it was observed that for all regions of the foot, except for the metatarsals, with the increase in the pressure value, the difference in measurements tends to be positive, meaning that under these conditions, the pedar^®^ insole registered higher values than the pneumatic insole. Even so, the lower and upper limits are close, and the average bias is very close to zero for all regions of the foot, which confers excellent levels of agreement: 91.2% for the toes region, 93.4% for the metatarsals, 94.9% for the heel, and 96.6% in the lateral-midfoot region. A closer analysis of these results shows that in some regions, evident clusters of values are formed for pressures at the extremes, sometimes for lower pressures, sometimes for higher pressures. This characteristic is more evident in the lateral-midfoot region, and the formation of these two clusters is due to moments of load transition during gait. For example, the cluster for the highest pressure is mainly formed at the body weight first impact instance in that air cell, or in the propulsion phase (for example, in the toes region). This sequence of events, which begins at the heel, is then propagated through all the plantar regions of the foot, although with different pressure magnitudes. The cluster for lower pressures is mainly formed during the plantar support phase, where the pressures are distributed and the intensity, which could be concentrated in a single region, is now starting to be reduced due to the pressure redistribution throughout the plantar region. For this cluster, there is also the contribution of the drastic reduction in pressure when the foot is in the lift-off at the moment of load transition between the two feet during gait.

## 5. Discussion

The results obtained in static and dynamic tests demonstrate the effectiveness of the pneumatic insole in the redistribution and plantar pressure offloading and the feasibility of the proposed solution. The statistical analysis revealed significant differences between the test conditions, proving the ability of the variable pressure system to modify the distribution of plantar pressures. In the static test, it was found that the pressure stabilization mechanism acted by redistributing the pressures, significantly reducing the pressure on the heel (32.43%) and redistributing part of this load to the toes and metatarsals. This result is consistent with what was expected, as the pressure distribution is limited by total body weight and can only be redistributed between different regions of the foot. The pressure offloading intervention proved to be even more effective, achieving a reduction of up to 42.72% in the heel when preceded by the initial pressure stabilization. This indicates that initial stabilization improves the effectiveness of the offloading by preparing a more homogeneous distribution of pressure before its reduction directed to a given region under static conditions.

In dynamic tests, the nature of the gait resulted in greater data variability, as evidenced by the high standard deviations. The pressure offloading at the heel achieved less significant reductions than in the static test when the stabilization process is not performed. When stabilization is performed, preceded by the offloading event, the pressure reduction can reach 24.73%. The data suggests that initial pressure stabilization may be a relevant factor, although in dynamic mode, the impact of walking may mask the effect of pressure redistribution.

The evaluation of the peak pressures confirmed that the intervention of the pneumatic insole not only reduced the peak pressure in the heel but also decreased the intensity of the peaks in other regions, such as metatarsals and toes, demonstrating an overall effect in reducing excessive pressures. However, the increased pressure in the midfoot region indicates that this area has absorbed part of the redistributed load, which may be a relevant factor in preventing injuries in regions more prone to overload, such as the heel and metatarsals. It should be noted that the pressure offloading intervention was only carried out in a single moment, and, in any daily use of the insole, this procedure can be performed several times until the desired pressure level is reached, without impairing the appropriate pressure pattern of the other regions of the foot. It is also worth mentioning that this procedure can only be performed until the air cell reaches the minimum air volume limit so that it remains operational and can make accurate pressure measurements.

The validation of the pneumatic insole using the Bland–Altman method demonstrated a high agreement (from 91% to 98%, minimum and maximum) between the values measured by the pedar^®^ and the pneumatic insole, in both tests and for all plantar regions. The results indicate that the pneumatic insole has a performance very close to that of the pedar^®^ insole, considered as the reference measurement system in the market. However, some systematic bias patterns have been observed, particularly at times of low pressure, where the pedar^®^ insole has limitations in the measurement of values lower than 15 kPa. In the dynamic test, the formation of clusters in the graphs indicates a direct relationship between the moments of gait transition and the variations in plantar pressure, reinforcing the importance of considering the gait cycle in the analysis of plantar pressure.

When compared with the study by Erel et al. [16], our approach introduces several benefits. In the existing study, an insole composed of a set of air cells that operate through inflation and deflation cycles was reported, in an approach inspired by anti-bedsore mattresses, with the aim of relieving plantar pressure. Although this concept allows localized pressure relief through alternating support, it has some limitations. In particular, it does not have real-time response or control based on clinical thresholds (such as the value of 200 kPa or 25% baseline pressure reduction [5]), often associated with an increased risk of ulceration in diabetic foot pathology. Additionally, the system proposed by Erel et al. was tested on open-toe sandals, which do not reflect real walking conditions or the typical load support of closed footwear. This limitation may compromise the external validity of the results and restrict the practical applicability of the solution for everyday use, a weakness recognized by the authors themselves. In contrast, the pneumatic insole developed in the present study was tested fully integrated into a shoe certified as a medical device, commonly used daily by people with diabetic foot, ensuring greater proximity to the user’s real conditions. This solution is prepared to operate continuously and autonomously, as it is configured as a portable device. It monitors plantar pressure in real-time and actively triggers air volume modulation in response to a predefined threshold, allowing dynamic, adaptive, and personalized pressure redistribution for each user. In our tests, both static and dynamic, it was possible to quantitatively demonstrate (Table 3 and Table 4) that pressure offloading (simulated event in the heel cell) resulted in pressure reductions of approximately 25% or more during the stabilization and offloading events.

Unlike Erel’s solution, which works by periodically circulating air without relying on continuous measurements of localized pressure, our system avoids unnecessary actions that may occur when a cell is unnecessarily depressurized, possibly ignoring areas where there is overpressure. Our approach allows simultaneous monitoring of the offload and eventual pressure increase in other cells, maintaining a balanced and controlled redistribution pattern, with the aim of preventing the creation of new overload points. This is evident in the results presented in Table 3 and Table 4, where the simulated pressure offloading event in the heel led to a redistribution of plantar pressure, resulting in a more uniform pressure distribution across all cells. Our system demonstrated a significant pressure reduction in the intervened regions, both in static and dynamic conditions. These design features, combined with the high accuracy of the measurements obtained, reinforce the potential of our solution as an effective tool for active monitoring and intelligent management of plantar pressure. Together, these features represent a significant advancement in the development of intelligent offloading systems tailored to the needs of high-risk patients.

### Challenges and Constraints

Despite the advances achieved with the development of this variable pressure system, some limitations and constraints inherent to this study must be considered. One of the main limitations is that the pedar^®^ system only measures pressures above 15 kPa, which can have some impact on the accuracy of measurements in regions where pressures are typically lower, such as the toes region in static mode. Thus, some type of error may be introduced in the measurements obtained, even if small. One constraint, although it has been easily overcome, is due to the acquisition rate of the two measurement systems used. The pedar^®^ system operates with a frequency of 100 Hz, while the pneumatic insole has an acquisition rate of 15 Hz, which requires the downsampling process to align the data obtained by the two systems at each time instant.

The reliance of this study on data obtained from a single participant also represents a limitation. Although this approach is acceptable for this stage of prototype development, it should be noted that not all findings can be generalized. Although the system’s accuracy and its pressure monitoring performance capacity have been demonstrated, it should be considered that the results of the pressure redistribution process and the reduction in peak plantar pressure may be influenced by user-specific factors, such as body weight, gait, or foot type.

Regarding the physical properties of the pneumatic insole material, no experimental tests have been carried out to evaluate its long-term elasticity, resistance, or deformation. However, the values provided by the manufacturers suggest that the materials used are suitable to withstand the intended usage conditions. Finally, a significant constraint of this study was the reliance on insole manual fabrication rather than industrial manufacturing processes. Implementation of pressurized injection molding techniques in a factory setting would enhance product quality, uniformity, and replicability. However, the time and costs associated with the preparation of an industrial production process were not compatible with the current developmental stage. Future iterations should prioritize transitioning to standardized manufacturing methods to ensure consistent performance across all produced insoles, while covering different sizes or shape adaptations.

## 6. Conclusions

In this study, a novel variable pressure system for monitoring and redistributing plantar pressure inside the shoe is presented, differing from conventional approaches. The central innovation of this variable pressure system, and in particular of the pneumatic insole, lies in its real-time monitoring capability, which allows a dynamic adjustment during the user’s daily activity. This is particularly important because, as identified in the literature, many of the existing solutions for ulcer prevention are passive and unable to adapt to changes in plantar pressures during the user’s daily occupations. Furthermore, the proposed system, by monitoring and responding automatically, ensures greater comfort and safety both for populations at higher risk, such as people with diabetes, but also for workers subject to high plantar stress and in physically exhausting environments.

In summary, the results of this study demonstrated quantitatively that the pneumatic insole was effective in redistributing plantar pressure, reducing excessive loads, and mitigating plantar peak pressures in critical areas under both static and dynamic conditions. Additionally, the validation of the system with the gold standard showed that the pneumatic insole is a reliable and suitable device for applications in the analysis of plantar pressure. The device shows potential for real-time monitoring and targeted interventions across clinical settings, athletic performance optimization, and preventive occupational health applications, offering a versatile, cost-effective solution where precise pressure management is crucial. Moreover, its portability and modularity make it a suitable platform for future research in the field of smart footwear and plantar pressure modulation. The system also demonstrates immediate applicability in clinical settings, particularly for ulcer prevention, due to the possibility of responding to personalized thresholds for pressure relief in diabetic foot care.

### Future Directions

Considering the results obtained in this study, there are several future research directions that can be explored to optimize and expand the applicability of the developed variable pressure system. One promising possibility is its integration into safety footwear, where it could contribute to improving the occupational safety and health conditions of workers subjected to long-standing periods or high levels of plantar loading. In addition, more comprehensive tests will be carried out to validate the effectiveness of the system in different population groups with a larger sample size and investigate its applicability in the prevention of plantar complications. Long-term fatigue analysis and deformation tests of the pneumatic insole materials will also be considered. These studies will consolidate the potential of the proposed system as an innovative and functional alternative for plantar pressure management, and also confirm its viability for daily use.

## Figures and Tables

**Table 1:** Prerequisites for manufacturing the variable pressure system.

Prerequisite	Description
Uniform redistribution of plantar pressure	The system must be able to monitor plantar pressure in real-time and, based on this data and at a certain threshold, actively redistribute pressure to prevent injuries, improve comfort, and help with rehabilitation, especially in cases of diabetic foot ^1^.
Maximum pressure threshold	A maximum pressure threshold of 200 kPa is the most consensual value described in the literature to avoid plantar overloads and injuries in people with diabetic foot [5,21]. The system must ensure, through a dedicated algorithm, that a certain pressure pattern above this threshold must activate the plantar pressure redistribution system. This threshold can also be customized and defined according to the user’s needs and under the instructions of their attending physician [5] ^1^.
Pressure adjustment mechanism	The system must include a pneumatic mechanism capable of actively actuating and redistributing plantar pressure in response to measurements taken, through air cells in an insole made of deformable rubber materials ^1,2^. The pressure redistribution mechanism must respond to the algorithm conditions and the pre-established pressure threshold [5,21].
Technical specifications	Coverage areas of the plantar region: heel, lateral-midfoot, metatarsals, and toes [4,13]. Pressure range: 1 kPa to 1000 kPa [5,13]. Resolution: 1 kPa ^1^. Pneumatic control: pressure sensors and solenoid valves [15,16,17]. Autonomy: battery with minimum autonomy for 8 h of continuous use [6] ^1^. Interface and data collection: smartphone app [6] ^1^. Connectivity: Bluetooth Low Energy (BLE) [6].
Usability	Portability: the pneumatic control unit must be portable so that it can be used throughout the day. Its attachment to the waist, for example, must not interfere with the user’s mobility ^1^. Comfort and integration: the insole must be comfortable, flexible enough, and, if possible, able to be attached to different types of footwear, without compromising the effectiveness of the system ^2^. Ergonomics: the integration of the system into the footwear must be ergonomic, without compromising the comfort or functionality of the foot while walking ^1,2^. Safety: the system must be safe for the user, with protection against electrical failures and with emergency mechanisms that allow it to be switched off ^1,2^.
Stability and durability	The materials used, such as pneumatic tubes, air cell structures, and other mechanisms, must be strong and durable enough to withstand the pressure involved ^2^.
User interface	It should be carried out through a mobile application with a simple and intuitive interface so that the user (or physician) can monitor the conditions of the system and adjust parameters without technical complications. However, at this stage of prototyping, a basic commercial application already available can be used to record the measurements obtained. Later, the data analysis can be carried out [6] ^1^.

^1^ Also based on health professionals; ^2^ also based on footwear experts.

**Table 2:** Characteristics of the materials used in the molding process to produce the insole structure.

Structure	Intermediate Pad	Insole with Air Cells
**Material**	Liquid silicone,	Liquid urethane rubber,
	Ecoflex™ 00-50, Smooth-On	VytaFlex™ 40, Smooth-On
**Application technique**	Pouring	Pouring and low-pressure injection
**Shore hardness scale** ^1^	00-50	40 A
**Tensile strength** ^2^	≈2.2 MPa	≈3.6 MPa
**Elongation to break** ^2^	980%	660%

^1^ ASTM D2240 standard; ^2^ ASTM D412 standard.

**Table 3:** Average interface pressure (AIP) recorded by the pedar^®^ insole in the static tests before and after the pneumatic insole interventions. The pressure stabilization process was carried out for all regions of the foot, and the offloading only at the heel.

Effect	Status		Foot Zones	Average Interface Pressure AIP ± SD (kPa)		Variation Before vs. After		*p*-Value
	Before	After		Before	After	(kPa)	(%)	
Stabilize	Not stabilized	Stabilized	Heel:	49.58 ± 3.99	33.50 ± 0.76	−16.08	−32.43	<0.001
			Midfoot:	20.48 ± 2.47	21.09 ± 0.52	0.61	3.00	<0.001
			Metatarsals:	11.39 ± 1.56	14.52 ± 0.29	3.13	27.53	<0.001
			Toes:	2.51 ± 0.37	3.69 ± 0.46	1.18	46.98	<0.001
Offload	Not stabilized	Not stabilized and offloaded	Heel:	52.81 ± 0.90	45.36 ± 1.06	−7.45	−14.10	<0.001
			Midfoot:	18.88 ± 0.57	22.07 ± 0.88	3.18	16.86	<0.001
			Metatarsals:	12.23 ± 0.38	13.36 ± 0.42	1.13	9.22	<0.001
			Toes:	1.28 ± 0.14	1.36 ± 0.15	0.08	6.44	<0.001
Offload	Stabilized	Stabilized and offloaded	Heel:	36.79 ± 0.91	26.20 ± 1.67	−10.19	−27.69	<0.001
			Midfoot:	19.92 ± 0.49	21.28 ± 0.57	1.36	6.81	<0.001
			Metatarsals:	15.31 ± 0.32	16.87 ± 0.64	1.56	10.18	<0.001
			Toes:	4.86 ± 0.29	5.01 ± 0.59	0.15	3.08	<0.001
Stabilize and offload	Not stabilized and offloaded ^1^	Stabilized and offloaded ^1^	Heel:	45.36 ± 1.06	25.98 ± 1.16	−19.38	−42.72	<0.001
			Midfoot:	22.07 ± 0.88	21.25 ± 0.63	−0.81	−3.69	<0.001
			Metatarsals:	13.36 ± 0.42	17.11 ± 0.55	3.75	28.07	<0.001
			Toes:	1.36 ± 0.15	5.15 ± 0.65	3.79	278.11	<0.001

^1^ Additional test to verify if stabilization process influences pressure offloading. Note: negative values indicate a reduction.

**Table 4:** Average interface pressure (AIP) recorded by the pedar^®^ insole in the dynamic tests before and after the pneumatic insole interventions. The pressure stabilization process was carried out for all regions of the foot, and the offloading only at the heel.

Effect	Status		Foot Zones	Average Interface Pressure AIP ± SD (kPa)		Variation Before vs. After		*p*-Value
	Before	After		Before	After	(kPa)	(%)	
Offload	Not stabilized	Not stabilized and offloaded	Heel:	77.11 ± 32.19	75.56 ± 26.25	−1.55	−2.00	<0.001
			Midfoot:	37.93 ± 9.56	41.41 ± 9.37	3.48	9.18	<0.001
			Metatarsals:	44.07 ± 19.30	43.89 ± 18.49	−0.18	−0.40	0.7055 ^1^
			Toes:	42.69 ± 15.74	38.53 ± 16.17	−4.16	−9.74	<0.001
Offload	Stabilized	Stabilized and offloaded	Heel:	91.49 ± 28.36	68.86 ± 32.42	−22.62	−24.73	<0.001
			Midfoot:	43.31 ± 9.47	42.33 ± 12.70	−0.97	−2.24	0.1343 ^1^
			Metatarsals:	39.36 ± 19.60	53.57 ± 17.44	14.21	36.10	<0.001
			Toes:	38.87 ± 20.42	51.65 ± 17.82	12.77	32.86	<0.001

^1^ Not significant, *p*-value > 0.01. Note: negative values indicate a reduction.

## Data Availability

The original data and contributions presented in the study are included in this article, but additional information can be requested from the corresponding author.

## Abbreviations

The following abbreviations are used in this manuscript:

AIP	Average interface pressure
BLE	Bluetooth Low Energy
CAD	Computer-aided design
FDM	Fused deposition modeling
FSS	Full Scale Span
IMU	Inertial measurement unit
IP	Interface pressure
PCB	Printed circuit board
PLA	Polylactic Acid
PP	Peak pressure
PV	Process value
SD	Standard deviation
SV	Setpoint value
